# Developing influenza and respiratory syncytial virus activity thresholds for syndromic surveillance in England

**DOI:** 10.1017/S0950268819000542

**Published:** 2019-03-26

**Authors:** S. E. Harcourt, R. A. Morbey, G. E. Smith, P. Loveridge, H. K. Green, R. Pebody, J. Rutter, F. A. Yeates, G. Stuttard, A. J. Elliot

**Affiliations:** 1 Real-time Syndromic Surveillance Team, Field Service, National Infection Service, Public Health England, Birmingham, UK; 2 Respiratory Diseases Department, National Infection Service, Public Health England, London, UK; 3 NHS Pathways, NHS Digital, Leeds, UK; 4 Advanced, Ashford, UK; 5 NHS England, Birmingham, UK

**Keywords:** Influenza, moving epidemic method, respiratory syncytial virus, syndromic surveillance, thresholds

## Abstract

Influenza and respiratory syncytial virus (RSV) are common causes of respiratory tract infections and place a burden on health services each winter. Systems to describe the timing and intensity of such activity will improve the public health response and deployment of interventions to these pressures. Here we develop early warning and activity intensity thresholds for monitoring influenza and RSV using two novel data sources: general practitioner out-of-hours consultations (GP OOH) and telehealth calls (NHS 111). Moving Epidemic Method (MEM) thresholds were developed for winter 2017–2018. The NHS 111 cold/flu threshold was breached several weeks in advance of other systems. The NHS 111 RSV epidemic threshold was breached in week 41, in advance of RSV laboratory reporting. Combining the use of MEM thresholds with daily monitoring of NHS 111 and GP OOH syndromic surveillance systems provides the potential to alert to threshold breaches in real-time. An advantage of using thresholds across different health systems is the ability to capture a range of healthcare-seeking behaviour, which may reflect differences in disease severity. This study also provides a quantifiable measure of seasonal RSV activity, which contributes to our understanding of RSV activity in advance of the potential introduction of new RSV vaccines.

## Introduction

Influenza and respiratory syncytial virus (RSV) are common viral causes of respiratory tract infections, with epidemics occurring every winter in temperate climates. Seasonal increases in influenza activity occur mostly in the winter months and are the subject of enhanced monitoring and surveillance in England to determine the timing and intensity of activity [1]. In healthy individuals, the resulting disease is usually self-limiting, with most patients recovering within a week. The greatest burden of illness is seen in children; however, populations such as the elderly, children under 6 months old, pregnant women and people with chronic conditions or immunosuppression are at increased risk of complications of influenza and have the greatest burden of serious illness [2–4].

RSV results in mild infections in adults and children but more severe disease presents in young infants and the elderly, including bronchitis and bronchiolitis in infants [5]. Research has shown that the burden of RSV is similar to or even exceeds that of influenza and therefore plays a significant role in pressures placed upon healthcare services [6–11].

As influenza and RSV can cause substantial mortality and morbidity, the ability to monitor the onset and intensity of community-based activity of both pathogens in near real-time is a key objective for winter pathogen surveillance [12]. Thresholds for monitoring influenza activity in the community have been developed for use with syndromic surveillance systems to indicate the onset of influenza virus circulation, to provide early warning and to give some indication of the intensity of influenza in the community [13–16]. In recent years, a variety of methodologies have been developed to create early warning and activity thresholds for influenza surveillance. These have included regression-based models and linear models largely based on time-series methods [14, 17, 18]. The Moving Epidemic Method (MEM) has been adopted across Europe as the standard methodology for classifying influenza activity using primary care consultation rates [17]. Using historical data, MEM calculates the timing and duration of an influenza epidemic by determining the minimum number of weeks with the maximum cumulative rate. Influenza activity rates that occur either side of these epidemic periods are defined as baseline activity (i.e. pre- and post-epidemic activity) and are used to calculate the epidemic threshold [17]. When primary care influenza-like illness (ILI) consultation rates cross the epidemic threshold, this then denotes the beginning of significant influenza activity in the community [17, 19]. Subsequent MEM thresholds (medium, high and very high) identify increasing intensity of influenza activity throughout the season, categorizing activity as ‘low to medium intensity’, ‘medium to high intensity’, ‘high to very high intensity’ and ‘above very high intensity’.

The aim of this paper is to evaluate the use of MEM with syndromic surveillance data in England derived from two novel syndromic surveillance data sources: GP unscheduled/out-of-hours consultations and telehealth call data. We also evaluate the innovative use of MEM for monitoring RSV activity in children aged under 5 years and the use of MEM for daily monitoring as part of a routine, national, syndromic surveillance service.

## Methods

### Syndromic indicators

Syndromic surveillance is the real-time (or near real-time) collection, analysis, interpretation and dissemination of health-related data [20]. Public Health England (PHE) coordinates a suite of national syndromic surveillance systems which monitor scheduled/in-hours and unscheduled/out-of-hours general practitioner (GP) consultations, attendances at sentinel emergency departments and calls to a National Health Service (NHS) telephone triage, healthcare advice and information line (NHS 111) for the early identification of the impact (or absence of impact) of potential public health threats warranting effective public health action [21].

GP out-of-hours (GP OOH) consultations and NHS 111 calls were analysed as the percentage of consultations/calls relative to the total count of GP consultations or NHS 111 calls, respectively. In the PHE GP OOH syndromic surveillance system, syndromic indicators are analysed as the percentage of consultations relative to all contacts where the clinician has recorded a diagnosis/sign/symptom using a Read code (a coded thesaurus of clinical terms). Percentages were used to take account of unexpected fluctuations in daily coverage of both systems.

Each syndromic indicator selected for this study (Table 1) had been previously validated as a sensitive measure of either influenza or RSV activity, based on previous clinical, epidemiological or microbiological experience [10, 11, 13]. The individual syndromic indicators were based either on aggregates of clinical Read codes used by GPs to diagnose presenting symptoms of a patient (GP OOHSS) [22] or the endpoint symptom classifier selected by call handlers using a clinical triage system during a patient telehealth call (NHS 111) [23]. GP Read codes and NHS 111 presenting symptoms had been previously developed for each system to best describe GP out-of-hours consultations for ILI and bronchitis (GP OOH) and calls for cold/flu and cough (NHS 111).

**Table 1.** Syndromic indicators selected as sensitive measures of influenza and respiratory syncytial virus activity and years of historical data used to calculate 2017–2018 MEM epidemic and intensity thresholds

MEM threshold	System	Syndromic indicator		Historical data included
Influenza	NHS 111	Cold/flu	All ages	2013–2014 to 2016–2017
	GP OOH	Influenza-like illness	All ages	2011–2012 to 2016–2017
RSV	NHS 111	Cough	<5years	2013–2014 to 2016–2017
	GP OOH	Bronchitis	<5years	2011–2012 to 2016–2017

NHS, National Health Service; GP OOH, general practitioner out-of-hours; RSV, respiratory syncytial virus.

### Creation and validation of MEM thresholds

A set of MEM thresholds were developed for the 2017–2018 winter season for influenza and RSV. Developing MEM thresholds requires a recommended minimum of 5 years’ historical surveillance data [24].

The ‘original method’ of the standard MEM package in the ‘R’ statistical software programme was used to generate influenza and RSV epidemic thresholds and activity intensity thresholds for medium-, high- and very high-intensity activity according to previous published methods [17, 25, 26].

We used 7-day moving average (7-dma) values (using the 7 most recent data points) to represent the required weekly values needed to calculate the thresholds. This in turn enabled breaches of the thresholds to be monitored on a daily basis by comparing the current daily 7-dma with the thresholds during the routine daily surveillance process. Prior to implementing thresholds for 2017–2018, thresholds for the seasons 2015–2016 and 2016–2017 had been developed and tested (data not presented). Validation of the MEM thresholds for 2017–2018 involved comparison with ILI GP consultation MEM thresholds generated for existing GP ILI surveillance systems including: the GP in-hours (GPIH) surveillance system (GPIHSS) [21]; the Royal College of General Practitioners (RCGP) Weekly Returns Service [27]; and an epidemic MEM threshold developed for the PHE Respiratory DataMart influenza laboratory specimen positivity data [28, 29]. For NHS 111 cough calls and GP OOH bronchitis consultations in children aged under 5 years, we compared the timing of breaches of the MEM activity thresholds with information on the timing of increases in national laboratory confirmations of RSV using the PHE Respiratory DataMart system [1, 30, 31].

For both systems, the performance of the new MEM thresholds was monitored using the daily 7-dma percentage consultations (GP OOH) and calls (NHS 111).

Thresholds were assumed to be breached after three consecutive days occurring at the higher/lower level. Previous experience of using thresholds showed that 1- or 2-day breaches early in the influenza season were not unusual, and therefore 3 days was considered a pragmatic approach. PHE routinely monitors real-time syndromic surveillance data for influenza and RSV activity using daily data. The weeks in which daily NHS 111 or GP OOH activity crossed the activity threshold were noted and used for comparison with those systems monitoring activity on a weekly basis.

The thresholds for the 2017–2018 season were applied retrospectively to previous seasons (2015–2016 and 2016–2017) to compare how different patterns of seasonal influenza activity performed against the threshold values, and the timing of threshold breaches.

## Results

### Influenza MEM thresholds

**Fig. 1.**
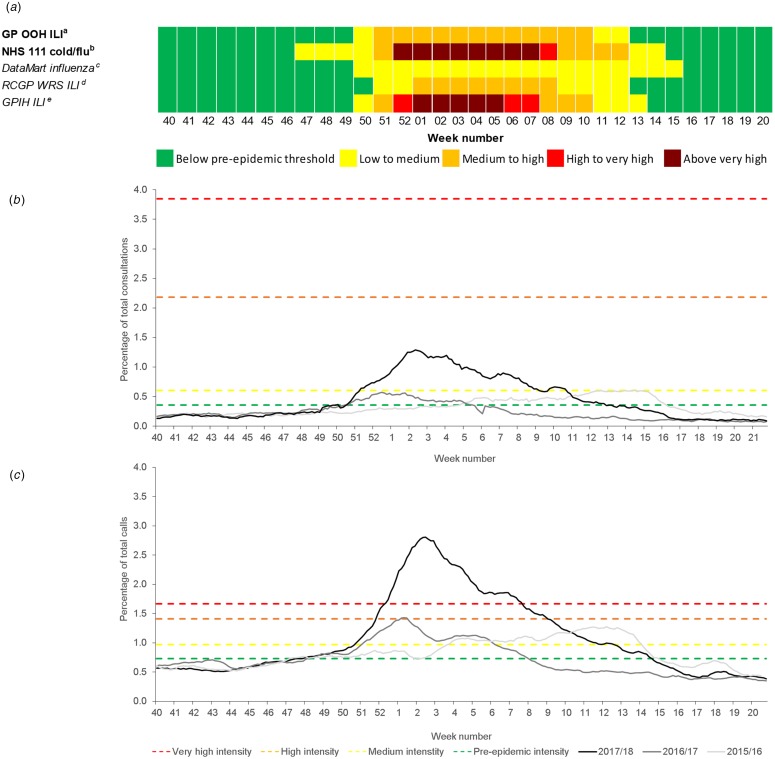
Syndromic surveillance indicators for influenza activity with 2017–2018 Moving Epidemic Method (MEM) thresholds: (a) summary of breaches of MEM influenza baseline epidemic and activity intensity thresholds during 2017–2018; (b) GP out-of-hours influenza-like illness consultations; and (c) NHS 111 cold/flu calls (both all ages). GP OOH, GP out-of-hours; ILI, influenza-like illness; NHS, National Health Service; RCGP WRS, Royal College of General Practitioners Weekly Returns Service; GPIH, GP in-hours. ^a^GP OOH consultations as a percentage of total consultations; ^b^NHS 111 calls as a percentage of total calls; ^c^Respiratory DataMart System (England) percentage of samples influenza-positive; ^d^RCGP WRS ILI consultation rate per 100 000 registered population; ^e^GPIH ILI consultation rate per 100 000 registered population.

The MEM thresholds for influenza were monitored prospectively for the 2017–2018 season (Table 2). The GP OOH ILI indicator breached the epidemic threshold in week 50/2017 and crossed the medium activity threshold for 12 weeks in week 51/2017 (Fig. 1a).

**Table 2.** Moving Epidemic Method (MEM) baseline epidemic and influenza/RSV activity intensity thresholds by syndromic system and indicator 2017–2018

		Syndromic indicator	2017–2018 Thresholds			
System			Baseline epidemic	Medium	High	Very high
NHS 111 *(percentage of calls)*	Influenza	Cold/flu *(all ages)*	0.73	0.97	1.41	1.66
	RSV	Cough *(under 5 years of age)*	10.61	14.52	17.39	18.83
GP OOH *(percentage of Read coded consultations)*	Influenza	Influenza-like illness *(all ages)*	0.36	0.60	2.18	3.85
	RSV	Bronchitis *(under 5 years of age)*	0.95	2.08	2.61	2.89
DataMart (*percentage positive*)	Influenza	Influenza	8.60	–	–	–
GPIHSS *(incidence rate per 100 000 population)*	Influenza	Influenza-like illness (all ages)	1.59	2.72	4.40	5.43
RCGP WRS *(incidence rate per 100 000 population)*	Influenza	Influenza-like illness (all ages)	13.10	24.20	68.70	108.90

NHS, National Health Service; GP OOH, general practitioner out-of-hours; RSV, respiratory syncytial virus; GPIHSS, general practitioner in-hours surveillance system; RCGP WRS, Royal College of General Practitioners Weekly Returns Service.

The NHS 111 cold/flu indicator was the first system indicator to breach the epidemic threshold in week 47/2017 and proceeded to reach very high activity during weeks 52/2017 to 7/2018, returning to baseline activity in week 15/2018 (Fig. 1a). The DataMart influenza epidemic threshold was breached between weeks 50 and 15; the GPIHSS epidemic threshold in week 50 (activity increased above the very high activity threshold in week 1 until week 5); and the RCGP epidemic threshold in week 51 (influenza activity remained in the medium category from weeks 1 to 8) (Fig. 1a).

NHS 111 cold/flu and GP OOH ILI activity in each of the years 2015–2016 and 2016–2017 was compared with the MEM activity thresholds calculated for the 2017–2018 season as a validation of the thresholds (Fig. 1b and c). Using the 2017–2018 MEM thresholds, NHS 111 cold/flu calls in 2015–2016 remained below the high-intensity threshold but would have breached this threshold in week 1/2017 (Fig. 1c).

### RSV MEM threshold (children under 5 years of age)

**Fig. 2.**
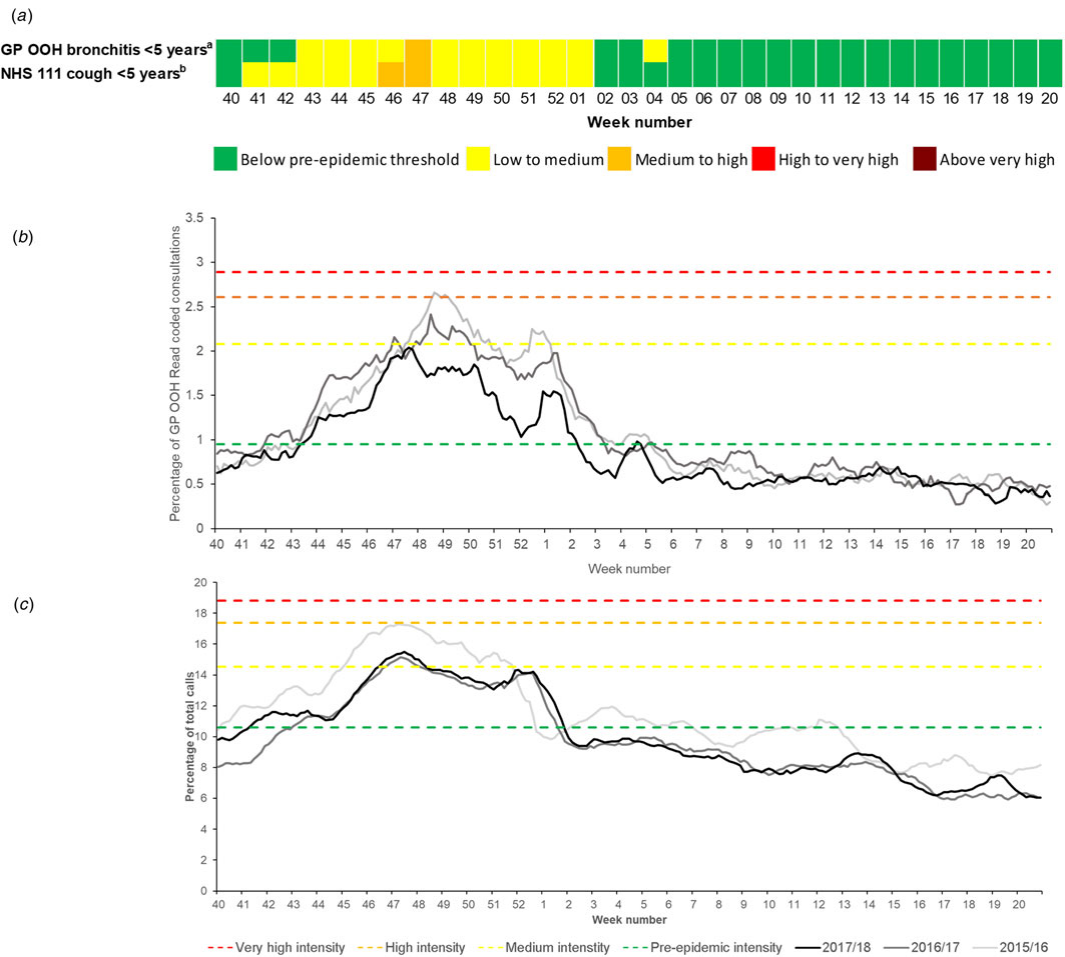
Syndromic surveillance indicators 2015–2018 for respiratory syncytial virus (RSV) activity with 2017–2018 Moving Epidemic Method (MEM) thresholds: (a) summary of breaches of MEM RSV baseline epidemic and activity intensity thresholds during 2017–2018; (b) GP out-of-hours bronchitis consultations; and (c) NHS 111 cough calls (both in children aged under 5 years). GP OOH, GP out-of-hours; ILI, influenza-like illness; NHS, National Health Service. ^a^GP OOH consultations as a percentage of total consultations; ^b^NHS 111 calls as a percentage of total calls.

In 2017–2018, the NHS 111 cough medium-intensity threshold of 14.5% was exceeded in weeks 46 and 47/2017 and GP OOH bronchitis activity breached the medium activity threshold (2.08%) for 1 week in week 47/2017 (Fig. 2a).

GP OOH bronchitis and NHS 111 cough activity in each of the years 2015–2016 and 2016–2017 were compared with the MEM activity thresholds calculated for the 2017–2018 season (Fig. 2b and c) and the percentage of RSV-positive laboratory reports for children under 5 years available from the PHE Respiratory DataMart system to further validate the thresholds [1, 30, 31]. Applying the 2017–2018 thresholds to 2015–2016, the NHS 111 cough indicator would have reached the high-intensity threshold in week 47, 3 weeks before the reported peak in the DataMart percentage RSV-positive laboratory reports in week 50/2015 (51%) [31].

In 2016–2017, there was a peak reported in the Respiratory DataMart percentage RSV-positive laboratory specimens (50%) in this age group in week 47/2016 [30]. This peak coincided with a peak in NHS 111 cough calls and an early peak in GP OOH bronchitis activity. However, the GP OOH bronchitis seasonal peak occurred later in week 48 and the activity remained in the medium- to high-intensity category until week 50/2016 (Fig. 2).

## Discussion

To date, the use of MEM activity thresholds has primarily been applied to primary care/GP consultation data for ILI [17]. However, MEM has also been applied to other types of data, such as acute respiratory infections [17], influenza laboratory reports [28, 32], pneumonia and influenza-related mortality [33], influenza-related hospitalisations [33] and staff influenza absenteeism data [34]. Here, we describe another innovative use of MEM using a wider range of syndromic surveillance respiratory indicators to develop thresholds for influenza and RSV surveillance, from novel public health surveillance data sources. National telehealth calls and GP OOH consultations were used to monitor daily activity and intensity of influenza and RSV as part of the national syndromic surveillance service.

### Main findings of this study

A set of thresholds were developed and applied prospectively to influenza surveillance data for the 2017–2018 season. The 2017–2018 influenza season was the most significant experienced in the UK since 2010–2011, with GP consultation rates for ILI and calls to NHS 111 peaking in week 2/2018 (Fig. 1) [1]. The NHS 111 cold/flu indicator breached the epidemic threshold in week 47, 3 weeks before all the other systems. It is possible that this early breach of the epidemic threshold may be due to the impact of other circulating respiratory pathogens. During 2017–2018, the NHS 111 cough and GP OOH bronchitis medium thresholds were breached in weeks 46 and 47, respectively, indicating an increasing intensity in RSV activity, which may have contributed to the NHS 111 cold/flu influenza epidemic threshold breach. This highlights the importance of monitoring intensity thresholds across different health systems to show differing impact.

The differences in the timing of the threshold breaches may reflect differences in the severity of the circulating influenza strains and differences in healthcare-seeking behaviour between different healthcare services. For example, in the UK, GP OOH and NHS 111 services are available for unscheduled health advice (in the evenings, weekends and public holidays), while routine in-hours GP services are not available at weekends or on public holidays, and often require a pre-requested appointment to see a GP. The impact of seasonal influenza on different age groups may also be a contributing factor to these observed differences. Influenza seasons where the elderly or very young are affected may impact on different health care services and thus syndromic surveillance systems. Therefore, the main advantage of using a suite of thresholds across several different health systems is the ability to capture a range of healthcare-seeking behaviour.

Another key finding of this study was the variation in intensity of activity measured by multiple system MEM thresholds during the 2017–2018 season, where the GP OOH ILI indicator remained at medium intensity, while the GPIH and NHS 111 moved into the very high threshold. This had the potential to impact on the public health messages produced, based on these results. It is likely that an underlying factor causing these discrepancies was the number of years of historical data included within the MEM threshold; GP OOH had two more years available, which may have increased the threshold values relative to current activity. This was also observed across other newly developed PHE influenza systems, e.g. the UK Severe Influenza Surveillance Systems (USISS) [1]. Guidance on MEM does dictate a minimum number of years of historical data that need to be included, however, in the case of newly developed systems, e.g. NHS 111, this can be problematic and multiple systems using different numbers of years can lead to results that are hard to interpret. As these systems continue to generate and collect more historical data, it will be possible to standardise the number of years of historical data used for threshold calculation across all the systems and standardise the inclusion/exclusion of future atypical years from the calculations.

RSV activity exhibits regular seasonal activity [35], with reported cases in children under 5 years increasing around mid-October (week 43) annually and peaking between weeks 47 and 52 [1]. It was therefore unsurprising that the RSV epidemic thresholds were breached by both syndromic surveillance systems early in the winter season. However, compared to RSV laboratory reporting, both the NHS 111 and GP OOH systems gave some early warning of the rise into medium-intensity activity levels. Therefore, despite the predictable seasonal activity of RSV, these results have shown some potential for providing some additional early warning of activity. However, of greater value is the ability to provide a quantifiable measure of RSV activity to compare intensity between seasons. In light of current developments of new RSV vaccines [36, 37], the ability to further describe RSV activity is a valuable resource that should be further explored.

### What is already known on this topic

Influenza early-warning thresholds have previously been applied to ILI, cold/flu and fever indicators in the PHE syndromic surveillance systems [13]. In the former PHE NHS Direct Syndromic Surveillance System, thresholds were developed using Poisson regression models based on the number of calls for cold/flu (all ages) and fever in 5–14 year olds [13]. Historically, syndromic surveillance systems have employed time-series and cumulative sum control chart (CUSUM) methods to construct early warning or activity thresholds [38]. MEM has been found to produce a robust and specific signal to detect influenza epidemics and has been shown to provide a good balance between the sensitivity and specificity of the epidemic threshold to detect seasonal epidemics and avoid false alerts [17]. In the UK, the MEM was shown to produce thresholds that were lower than traditionally used methods [19]. MEM thresholds were considered more appropriate indicators of the start of influenza virus circulation in the community, and the MEM approach was subsequently adopted by the constituent countries of the UK for the 2013–2014 season.

Using the MEM approach has allowed for the standardisation and harmonisation of ILI thresholds across 24 European countries and the UK and allows for comparison both between countries and across seasons [17]. This promotes understanding of epidemic patterns of seasonal epidemics and facilitates the evaluation of control measures such as vaccination campaigns [24]. In addition, the implementation of MEM to syndromic surveillance will improve cross-country comparison of influenza and RSV activity data at a time when the use of syndromic surveillance systems across Europe is expanding [20].

### What this study adds

We have developed a suite of influenza and RSV activity thresholds using novel data sources. Internationally, there are other examples of syndromic surveillance systems based on telehealth systems [39–41], but to our knowledge, this is the first time that such telehealth data have been used to construct influenza MEM thresholds. We have also used telehealth data to test MEM activity thresholds for specific age groups, e.g. for cough in children aged under 5, again demonstrating an innovative use of this methodology. The development of intensity thresholds for a range of different systems, indicators and age groups enables the monitoring of the impact of influenza and RSV on different components of the NHS.

The development of activity thresholds for bronchitis in children under 5 years is an innovative use of the MEM and will contribute to our understanding and monitoring of RSV in this age group. The development of future RSV vaccines will drive further requirements for enhanced RSV surveillance programmes to establish pre-vaccine morbidity baselines, describe the epidemiology of RSV activity and establish mechanisms for describing and comparing seasonal intensity to contribute to post-vaccine effectiveness work programmes [36, 37].

### Strengths

Established MEM thresholds use weekly data. One of the strengths of this current application of MEM is that the data are monitored daily as part of the routine PHE syndromic surveillance service. Using daily data, it is possible to pinpoint within each week the point at which an activity threshold has been crossed by daily monitoring of the 7-dma. This system is designed to alert to changes in the activity level once three consecutive days’ breach has been noted. The advantage of using daily data for monitoring using MEM is that it is more timely and enables reporting of a threshold breach in near real-time.

These new MEM activity thresholds are now incorporated into the routine outputs for these syndromic systems and will contribute to monitoring the timing of the start of influenza epidemics and their intensity in England. Using the MEM enables these thresholds to be compared with thresholds developed for other systems and data sources and will improve the ability to harmonise public health messages between systems and across different countries.

### Limitations

A limitation of our approach was the potential lack of specificity of syndromic indicators used for influenza surveillance compared with other indicators that had been based upon confirmed laboratory reports or sentinel GP ILI consultations. However, previous work has illustrated the close association between syndromic data and pathogen activity and therefore the impact of this was limited within the confines of this work [11, 42–44].

The observed differences in the timing of threshold breaches between systems could be explained by differences in healthcare-seeking behaviour and the severity of circulating strains of influenza/RSV but could also result from differences between the years included in the calculation of the baselines. This is a significant limitation but one which should diminish as more years of historical data become available. It should be possible in future years to standardise the years used to calculate baseline data across systems.

### Further work

As this work progresses, syndromic data collected during the 2017–2018 winter season will be incorporated into refreshed MEM thresholds for both the NHS 111 and GP OOH systems, and the thresholds used prospectively during the 2018–2019 and subsequent seasons [21].

The differences in timing of threshold breaches between different systems need further investigation to understand how each system contributes to the early warning of influenza and RSV in the community.
